# Comparative transcriptome analysis of the mechanism difference in heat stress response between *indica* rice cultivar “IR64” and *japonica* cultivar “Koshihikari” at the seedling stage

**DOI:** 10.3389/fgene.2023.1135577

**Published:** 2023-04-21

**Authors:** Yingfeng Wang, Yubo Wang, Wenjuan Chen, Yating Dong, Guilian Zhang, Huabing Deng, Xiong Liu, Xuedan Lu, Feng Wang, Guihua Chen, Yunhua Xiao, Wenbang Tang

**Affiliations:** ^1^Hunan Provincial Key Laboratory of Rice and Rapeseed Breeding for Disease Resistance, College of Agronomy, Hunan Agricultural University, Changsha, China; ^2^State Key Laboratory of Hybrid Rice, Hunan Hybrid Rice Research Center, Changsha, China

**Keywords:** rice (*Oryza sativa* L.), *indica*, *japonica*, heat stress, RNA-seq, DEGs

## Abstract

Heat stress (HS) has become a major abiotic stress in rice, considering the frequency and intensity of extreme hot weather. There is an urgent need to explore the differences in molecular mechanisms of HS tolerance in different cultivars, especially in *indica* and *japonica*. In this study, we investigated the transcriptome information of IR64 (*indica*, IR) and Koshihikari (*japonica*, Kos) in response to HS at the seedling stage. From the differentially expressed genes (DEGs) consistently expressed at six time points, 599 DEGs were identified that were co-expressed in both cultivars, as well as 945 and 1,180 DEGs that were specifically expressed in IR and Kos, respectively. The results of GO and KEGG analysis showed two different HS response pathways for IR and Kos. IR specifically expressed DEGs were mainly enriched in chloroplast-related pathways, whereas Kos specifically expressed DEGs were mainly enriched in endoplasmic reticulum and mitochondria-related pathways. Meanwhile, we highlighted the importance of NO biosynthesis genes, especially nitrate reductase genes, in the HS response of IR based on protein-protein interaction networks. In addition, we found that heat shock proteins and heat shock factors play very important roles in both cultivars. This study not only provides new insights into the differences in HS responses between different subspecies of rice, but also lays the foundation for future research on molecular mechanisms and breeding of heat-tolerant cultivars.

## 1 Introduction

Agriculture production is threatened by the frequent change of the climate, which directly influences food security. In the second half of the 20th century, the average global temperature increased by 0.74°C, and it is expected that the global temperature will increase at an average rate of 0.2°C/10a in the next 20–30 years (Jaagus, 2006). Climate warming has led to an increase in the intensity and frequency of extreme hot weather, and results in heat stress (HS), which causes irreversible damage to the growth and development of plants (Xu et al., 2021). As a widely planted crop, rice (*Oryza sativa* L.) is a staple food for over half of the population and faces a multitude of biotic and abiotic stresses continuously during their lifespan, including salt, temperature, drought and so on. HS is one of the major abiotic stresses that affects rice growth and development, which may occur in all the growing stages of rice (Tripathi et al., 2016; Shahzad et al., 2021). For every 1.0°C increase in average daily temperature during the growing season, the yields of rice will approximately be reduced by 14%, which poses a great threat to the cultivation of rice. Therefore, exploring the HS molecular response mechanism and mining beneficial genes to cultivate heat-tolerant cultivars represent an attractive approach to cope with this challenge (Kan et al., 2022).

HS influences physiological and metabolic processes of rice, which cause growth and development impaired. In terms of physiological effect, it is manifested as membrane damage, reactive oxygen species (ROS) accumulation, photosynthesis damage, carbohydrate metabolism and partition chaos, and phytohormone imbalance. As the most sensitive component of plant cells, the structure and function of membranes are easily damaged by HS. HS alters the ratio of saturated to unsaturated fatty acids by decreasing the activities of fatty acid desaturases, thereby disrupting the integrity of the plasma membrane, which in turn increases permeability and leads to leakage of organic and inorganic ions from cells (Fukao et al., 2019; Yang et al., 2019). HS makes the content of superoxide dismutase (SOD), catalase (CAT) and intracellular malondialdehyde (MDA) abnormal and impairs the activity of antioxidant enzymes and protein function. HS destroys the permeability of thylakoid membrane and even disintegrates thylakoid grana, triggering a decrease in chlorophyll content, which further leads to a decrease in the ratio of variable fluorescence to maximum fluorescence (Fv/Fm) and photosynthetic rate (Hu et al., 2014; Wang et al., 2017). HS affects the expression of carbohydrate metabolism-related genes such as carbon starved anthers (*CSA*), monosaccharide transporter (*MST8*), anther-specific cell wall invertase (*INV4*) and sucrose transporter (*OsSUT1*), which disturb the energy production, transport and distribution. Jasmonates (JAs), brassinosteroids (BRs), ethylene and abscisic acid (ABA) are major phytohormones that can act either synergistically or antagonistically to mediate HS through activating the defense system and enhancing osmotic regulation (Yang et al., 2021).

In the long-term co-evolutionary with HS, plants have evolved different coping mechanisms. On the one hand, plants can improve their ability to scavenge unfolded proteins by “actively response”, thereby maintaining protein homeostasis for heat resistance. On the other hand, plants can also be insensitive by “static braking” to reduce the consumption of thermal responses, maintain normal physiological activity, and rapidly rebuild after HS to improve survival under HS. Mining heat-resistance genes or quantitative trait loci (QTL) and elucidating the molecular mechanism are the key points for HS tolerance improvement in crops. So far, more than 80 rice HS-related QTLs have been mapped, 25 genes have been cloned, including 12 genes (*OsTT1、OsHTAS、TOGR1、ER、OsHCI1、OsHIRP1、OsSIZ1、OsNSUN2、MSD1、OsMDHAR4、OsUBP21、TT2*) encoding proteases, 10 genes (*OsHSP90、HSP101、OsANN1、OsRGB1、OsRab7、OsCNGC14、OsCNGC16、OsHSBP1、OsHSBP2、OsFBN1*) encoding heat shock proteins (HSPs), channel proteins and other proteins, and 3 genes (*OsMYB55、SNAC3、OsNTL3*) are TFs (Rana et al., 2012; Lim et al., 2013; Wei et al., 2013; Li et al., 2015; Wang et al., 2015; Wang et al., 2016; Kilasi et al., 2018; Zhou et al., 2019; Chen et al., 2021). These genes through complex molecular mechanism to HS response, including classic cognition (reactive oxygen species scavenging, cytotoxic protein elimination and unfolded protein renaturation), and newly regulatory pathway (G protein regulation, Ca^2+^ signal transduction and decoding and wax metabolism) (Penke et al., 2018).

During the seedling stage, the optimum growth temperature of rice is 26°C–32°C, HS (above 35°C) results in growth retardation, accelerated aging, decreased absorption capacity, and maybe even death (Liu et al., 2021). There is a pathway correlation between the basic heat tolerance at seedling stage and later growth stage of rice, which makes the research on the molecular mechanism of HS in seedling stage of great application valuable. Different genetic backgrounds have different responses to HS, especially between rice subspecies (*Indica* and *Japonica*) which show a great significant difference in enduring tolerance.

High-throughput RNA-sequencing technology (RNA-seq) provides highly accurate and cost-effective tools to unravel the transcriptome dynamics and molecular mechanisms of rice response to HS (Vitoriano and Calixto, 2021). The process of natural and artificial selection has endowed *indica* rice with greater heat tolerance than *japonica* rice (Xu et al., 2020). The *indica* cultivar IR64 (IR) is the parent of modern indica rice cultivars (Maeda et al., 2019). The *japonica* cultivar Koshihikari (Kos) is an elite Japanese cultivar. Both IR and Kos are the most popular *indica* and *japonica* rice cultivars in the world and are widely grown in various rice producing areas (Ebitani et al., 2005; Nonoue et al., 2019). In this study, we analyzed the leaf transcriptomes of IR and Kos at 0.5, 1, 2, 4, 8, 24 h of HS treatment and performed Illumina RNA-seq, transcriptome expression analysis and qRT-PCR to identify differentially expressed genes (DEGs) and pathway details that are responds to HS. The results of this study will not only enhance our understanding of the mechanisms by which plants respond to HS, but also provide new insights into the key genes responsible for the differences in HS tolerance between *indica* and *japonica*.

## 2 Materials and methods

### 2.1 Plant growth conditions and HS treatment

IR64 (IR) and Koshihikari (Kos) were used in this study. Seeds of both cultivars were cultured at 37°C for 2 d for germination. Germinated seeds were transferred to 96-well plates and seedlings were cultured in distilled water for 4 days, and then replaced with Kimura B nutrient solution. Before the stage of three-leaf, the temperature was 28°C. After the stage of three-leaf of two cultivars, the seedlings were transferred to HS treatment with a temperature of 45°C. Seedlings of both species were grown in an artificial incubator with a relative humidity of 70%. After HS treatment, the seedlings were recovered at 28°C.

Here, we selected seven time points (0, 0.5, 1, 2, 4, 8, and 24 h) to collect leaves of seedling for RNA-seq. A total of 42 samples were obtained in this study, with 0 h as the control group and 0.5, 1, 2, 4, 8 and 24 h as the treatment groups. There were three biological replicates for each group, and one biological replicate with 10 plants sampled. For each biological replicate, samples from each treatment were harvested separately and immediately frozen in liquid nitrogen and stored at −80°C for RNA extraction for transcriptome sequencing.

RNA extraction using the TRIzol method. RNA degradation and contamination was monitored on 1% agarose gels. Reverse transcription was performed using the PrimeScript RT kit (TakaRa, Dalian, China).

### 2.2 HS tolerance analysis of IR and Kos

After treatment at 45°C for 40 h and recovery for 3 days, the seedlings were photographed and the survival rate was counted, using seedlings grown under normal conditions in another incubator as a control. Electrolyte leakage from seedlings treated with HS for 24 h was measured using a previously described method (Dai et al., 2020).

### 2.3 Analysis of DEGs

HTseq (1.99.2) was used to statistically calculate the read count values of each gene, which represent the basic expression of gene, and standardize each gene expression was using fragments per kilobase of transcript per million mapped reads (FPKM), which makes the genes of two cultivars and groups comparable. DESeq (1.34.0) was using to screen the differential expression of genes with the conditions: an expression difference of |log_2_ Fold Change|>0 and a Significant padj<0.05.

Gene Ontology (GO) and Kyoto Encyclopedia of Genes and Genomes (KEGG) analyses of DEG were performed by the PlantNGSTools R package. Terms or entries with corrected *p*-values less than 0.05 were considered significantly enriched for DEGs. The sequences of transcription factors (TFs), transcriptional regulators (TRs) and protein kinases (PKs) were searched in the iTAK database (http://itak.feilab.net/cgi-bin/itak/index.cgi, accessed on 1 July 2022) and compared with differential gene sequences. SIRING (https://string-db.org/, accessed on 1 July 2022) is used to analyze protein-protein interaction (PPI) networks. Cytoscape v.3.9.0 was used to drawn the regulatory network of target genes.

### 2.4 Quantitative real-time PCR (qRT-PCR) validation of DEGs

Total RNA extraction and reverse transcription as described in 2.1. Ten DEGs were randomly selected for the verification of the RNA-seq result. The primers for these genes are listed in Supplementary Table S1. The qRT-PCR was performed in 96-well plates in 10 µL reaction using ChamQ™ SYBR^®^ Color qPCR Master Mix (Vazyme, Shanghai, China), *UBQ* gene was used as an internal control. Relative gene expression levels were determined from equation 2^−△△CT^. Three biological replicates were used for each gene under each time point.

## 3 Results

### 3.1 Phenotypes of two rice cultivars under HS treatment

**FIGURE 1:**
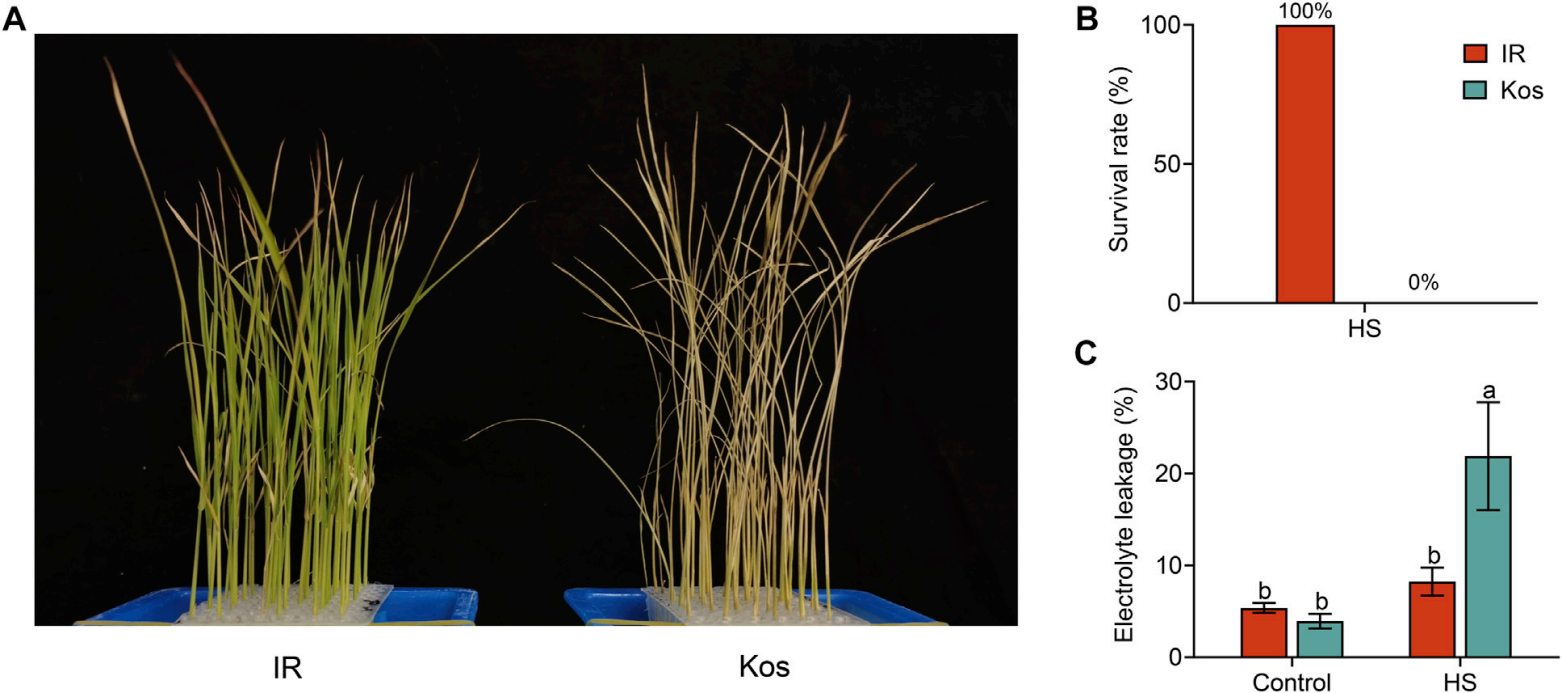
Phenotypes of two rice varieties subjected to a HS treatment. IR and Kos seedlings treated at 45°C for 40 h and recovered for 3 days **(A)**, and the survival rate of two rice varieties **(B)**. **(C)** Electrolyte leakage rate of IR and Kos seedlings after 24 h of high temperature treatment at 45°C. The corresponding seedlings grown under normal conditions in another chamber were used as controls. Survival rate in **(B)** is the proportion of surviving seedlings per 12 plants, and data are presented as mean ± SD, n = 4. The data shown in **(C)** are means ± SD of three independent experiments, and different letters indicate significant differences using ANOVA test, *p* < 0.05.

To assess the different responses of the two cultivars (IR and Kos) to HS, we transferred rice seedlings at the three-leaf stage to an artificial incubator for a HS treatment (45°C). After 40 h of treatment and 3 days of recovery at 28°C, all Kos seedlings died with 0% survival rate, whereas all IR seedlings survived (Figure 1A, B). Moreover, the electrolyte leakage of Kos seedlings after HS treatment was significantly higher than that of IR seedlings (Figure 1C). These results demonstrated that the *indica* rice cultivar IR from the tropics has stronger HS tolerance than the *japonica* rice cultivar Kos from the temperate zone, and showed extremely significant difference between rice subspecies.

### 3.2 RNA-seq results of transcriptome samples

To study the gene expression changes in IR and Kos leaves under HS as described above, we used next-generation sequencing technology to measure the transcription in rice leaves after 45°C HS treatment. RNA sequencing data were generated from rice leaf samples at different time points. The transcriptome of each cultivar was analyzed at seven time points with three biological replicates for each time point, and Illumina RNA-seq analysis of 42 samples yielded 355.25G bp of data and 2,425,820,766 read pairs (obtained reads) (Supplementary Table S2A). After re-processing of the reads, we obtained 2,368,378,416 single-end clean reads (total records) (Supplementary Table S2B). The total map ratio ranged from 89.82% to 95.98%, and the unique map ratio ranged from 87.45% to 93.16%, few reads could not be mapped to the reference genomes (4.02%–10.18%) (Supplementary Table S2B).

We used sample-to-sample correlation analysis for the data exploration analysis. The overall relatedness of the transcriptome at different times was determined by a correlation heatmaps generated for the IR and Kos samples (Supplementary Figure S1). For all samples, the three biological replicates (IR and Kos) showed good correlation, and the transcriptome data were closely related at each time point. These results indicate that the samples and transcriptome sequencing results from this study are available and can be analyzed further.

### 3.3 DEGs analysis and qRT-PCR verification

We analyzed the cultivar DEGs at different time points in order to identify DEGs involved in the regulation of HS in rice (Table 1). Comparing the DEGs of each cultivar to their control at different time points, the number of upregulated genes were greater than the downregulated genes. IR and Kos had the largest number of DEGs at 24 h after HS, with 13,724 (7,526 upregulated, 6,198 downregulated) and 15,808 (8,364 upregulated, 7,444 downregulated) DEGs, respectively. In IR and Kos, the lowest number of DEGs were identified 4 h after HS, with 5,199 and 5,470, respectively.

**TABLE 1:** Statistics of DEGs between two cultivars.

Combination	DEG set name	All	Up-	Down-
		DEGs	Regulated	Regulated
IR_0.5 h vs. IR_0 h	IR_0.5 h	6,270	3,874	2,396
IR_1 h vs. IR_0 h	IR_1 h	12349	6,513	5,836
IR_2 h vs. IR_0 h	IR_2 h	10043	5,516	4,527
IR_4 h vs. IR_0 h	IR_4 h	5,199	2,858	2,341
IR_8 h vs. IR_0 h	IR_8 h	12187	6,316	5,871
IR_24 h vs. IR_0 h	IR_24 h	13724	7,526	6,198
Kos_0.5 h vs. Kos_0 h	Kos_0.5 h	8,109	4,298	3,811
Kos_1 h vs. Kos_0 h	Kos_1 h	8,778	4,838	3,940
Kos_2 h vs. Kos_0 h	Kos_2 h	10003	5,188	4,815
Kos_4 h vs. Kos_0 h	Kos_4 h	5,470	3,002	2,468
Kos_8 h vs. Kos_0 h	Kos_8 h	9,033	4,829	4,204
Kos_24 h vs. Kos_0 h	Kos_24 h	15808	8,364	7,444

To verify the reliability and accuracy of the transcriptome results in this study, 10 DEGs were randomly selected and the expression levels of the two cultivars were verified by qRT-PCR at each time point after HS treatment. The qRT-PCR results for the 10 genes are shown in Supplementary Figure S2. The qRT-PCR results showed that the trends of these 10 genes after HS treatment were consistent with the transcriptome results, which indicated that our transcriptome results were reliable.

### 3.4 Determination of core DEGs in response to HS

**FIGURE 2:**
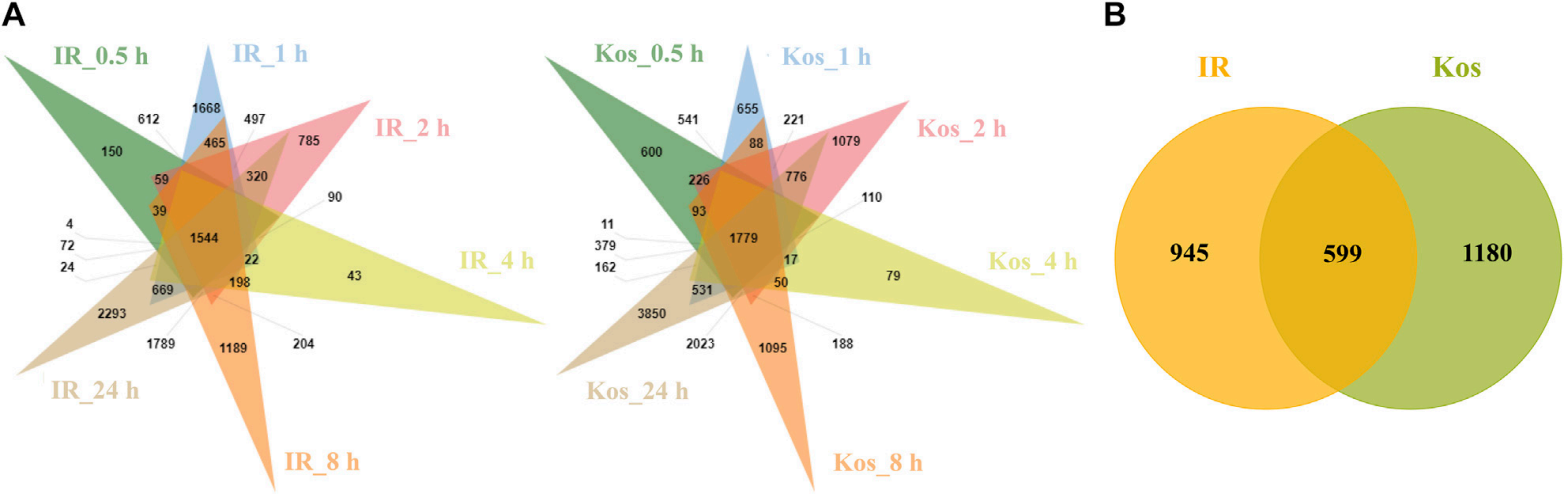
**(A)** Venn diagram of DEGs in both two cultivars at different time points after HS. IR_0.5 h, IR_1 h, IR_2 h, IR_4 h, IR_8 h, IR_24 h denote differentially expressed gene sets obtained by comparing IR samples at 0.5, 1, 2, 4, 8, and 24 h after HS, respectively, with the control sample IR_0 h. Kos_0.5 h, Kos_1 h, Kos_2 h, Kos_4 h, Kos_8 h, Kos_24 h denote differentially expressed gene sets obtained by comparing Kos samples at 0.5, 1, 2, 4, 8, and 24 h after HS, respectively, with the control sample Kos0. **(B)** Venn diagram of DEGs shared between both cultivars at all the time points after HS.

We first analyzed all time point DEGs for the two cultivars IR and Kos separately. After HS treatment, 1,544 and 1,779 DEGs were shown to be continuously expressed within six time points in IR and Kos leaves, respectively (Figure 2A). We then compared the continuously expressed DEGs in the two cultivars, and as shown in Figure 2B these DEGs were classified into three categories. There are 599 common (non-cultivar-specific) HS-responsive (CHR) DEGs, 945 IR HS-responsive (IHR) DEGs and 1,180 Kos HS-responsive (KHR) DEGs.

### 3.5 GO and KEGG enrichment analysis of IHR and KHR DEGs

**FIGURE 3:**
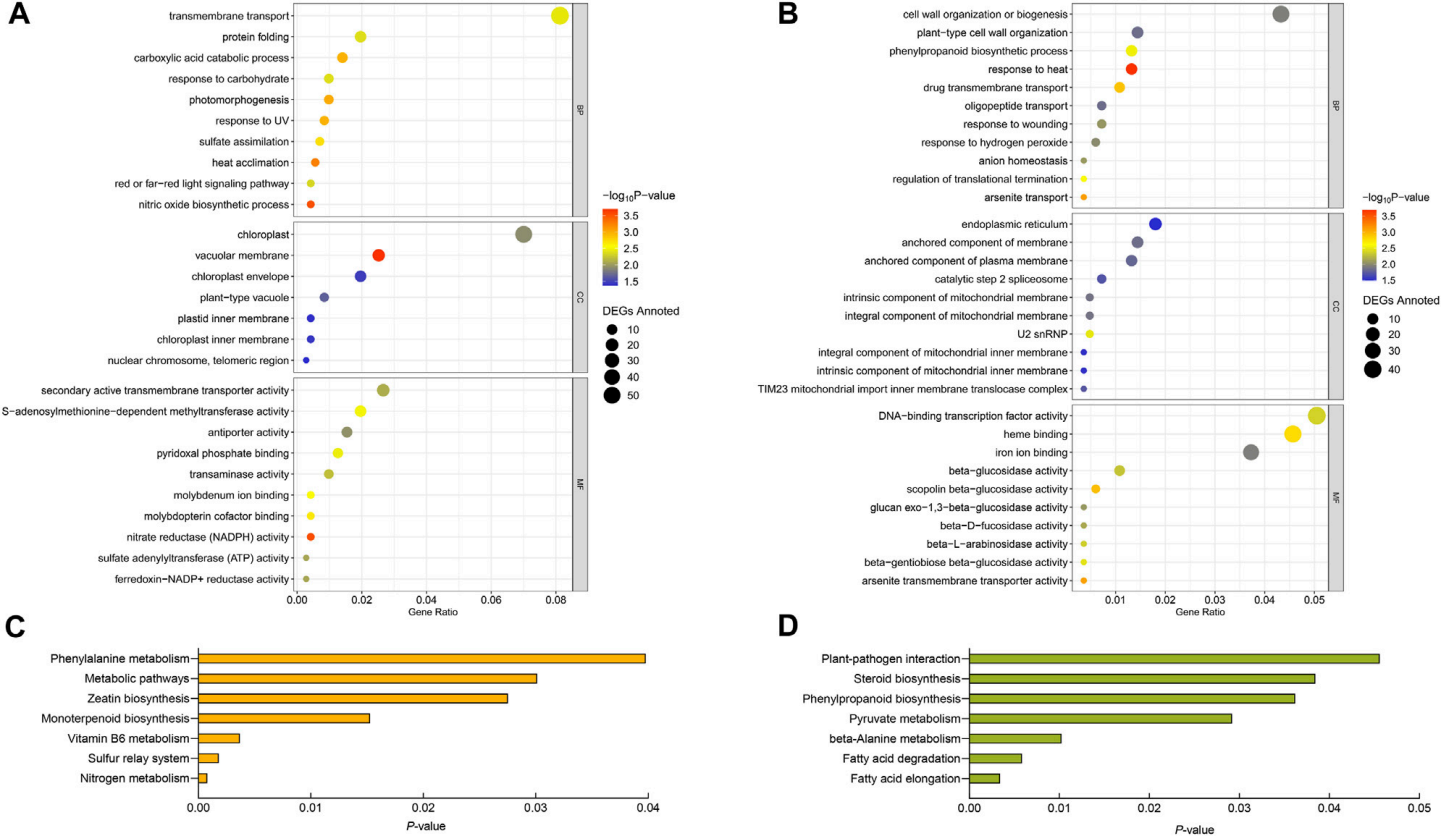
Analysis of GO and KEGG of IHR and KHR DEGs. GO analysis of IHR **(A)** and KHR **(B)** DEGs. Y-axis shows the GO terms. X-axis shows the Gene Ration. The size and color of each point represents the number of genes enriched in a particular GO term and the -log_10_ (*p*-value), respectively. KEGG pathway analysis of IHR **(C)** and KHR **(D)** DEGs. Y-axis shows the name of KEGG pathway. X-axis shows the *p*-values.

As shown in Figures 3A, B, the IHR and KHR DEGs annotated in the GO database were classified into three major categories including biological processes (BP), cellular component (CC), and molecular functions (MF), and the top ten terms with the smallest *p*-values in each category are shown by us in figure. The results revealed that the HS-response mechanisms were different in IR and Kos. For the selected 945 DEGs that were IHR, 714 had GO annotations and were involved in 132 terms. IHR DEGs are involve in transmembrane transport, component of chloroplast, heat acclimation, secondary active transmembrane transporter activity, nitric oxide biosynthetic process and others. For the selected 1,180 DEGs that were KHR, 832 had GO annotations and were involved in 94 terms. KHR DEGs involve response to heat, component of mitochondrial, beta-glucosidase and others.

To further investigate the DEGs involved in various pathways in IHR and KHR, KEGG enrichment analysis was performed for these two categories of DEGs. DEGs of IHR and KHR were enriched into 86 and 74 KEGG pathways, respectively, and we showed the KEGG pathways with *p*-values <0.05 in Figures 3C, D. The DEGs of IHR are involved in metabolic pathways, nitrogen metabolism and zeatin biosynthesis, among others, with the largest number of DEGs in metabolic pathways at 66. The DEGs of KHR are involved in fatty acid elongation and degradation, pyruvate and beta-alanine metabolism, etc. In addition, both categories contain pathways associated with phenylalanine.

### 3.6 Identification of TFs, TRs and PKs in IHR and KHR DEGs

**FIGURE 4:**
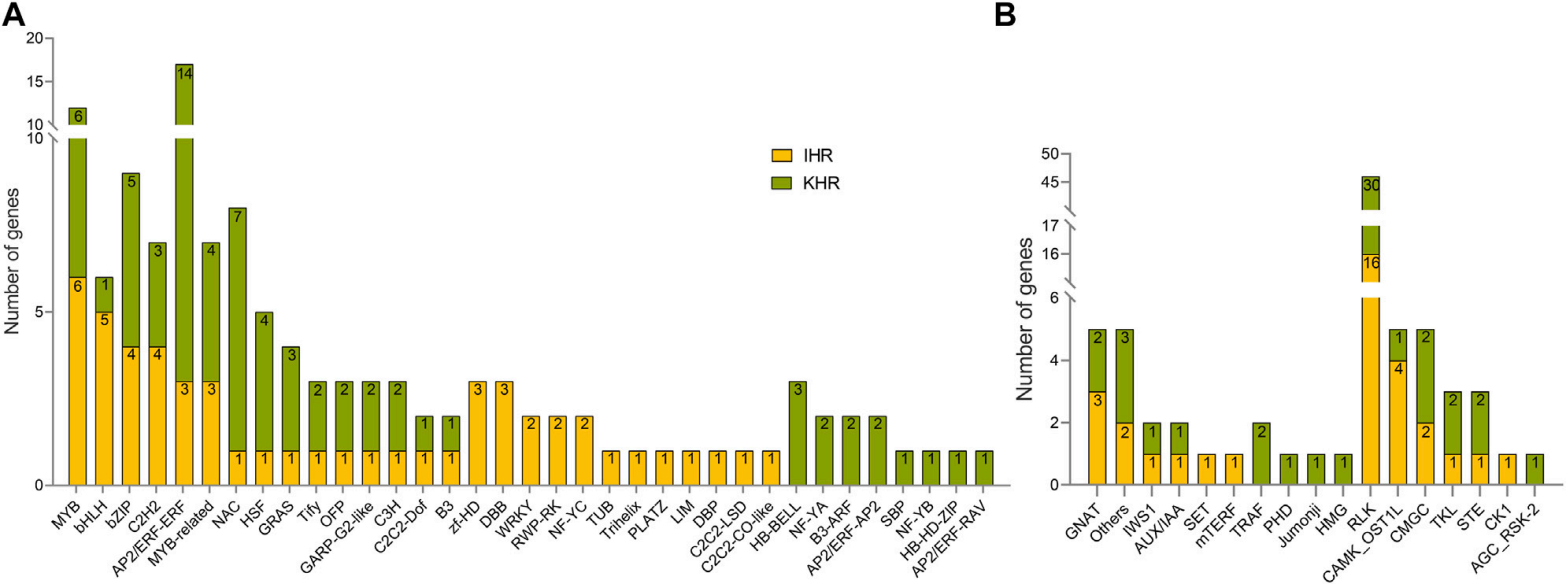
TF, TR and PK classification of IHR and KHR DEGs. **(A)** TF identification based on the iTAK database for DEGs of IHR and KHR, the numbers in the figure indicate the number of genes contained in each type of TF family. **(B)** The first 10 columns represent the TR identified in the IHR and the KHR. The latter 7 columns represent the PK identified in the IHR and the KHR. The numbers in the figure indicate the number of genes in TR and PK.

To better analyze the possible key genes of rice seedlings in response to HS, we identified TFs, TRs and PKs in IHR and KHR DEGs (Figure 4). As shown in Figure 4A, 53 and 77 TF genes were identified in the DEGs of IHR and KHR, respectively, which are contained in 36 TF families. In IHR, the TF family with the highest number of genes is MYB, while in KHR it is AP2/ERF-ERF. In addition, 10 TR families and 7 PK subfamilies were identified (Figure 4B). Among PKs, the majority of subfamilies belonged to the RLK (Figure 4B).

### 3.7 PPI network construction and core gene analysis of IHR and KHR DEGs

**FIGURE 5:**
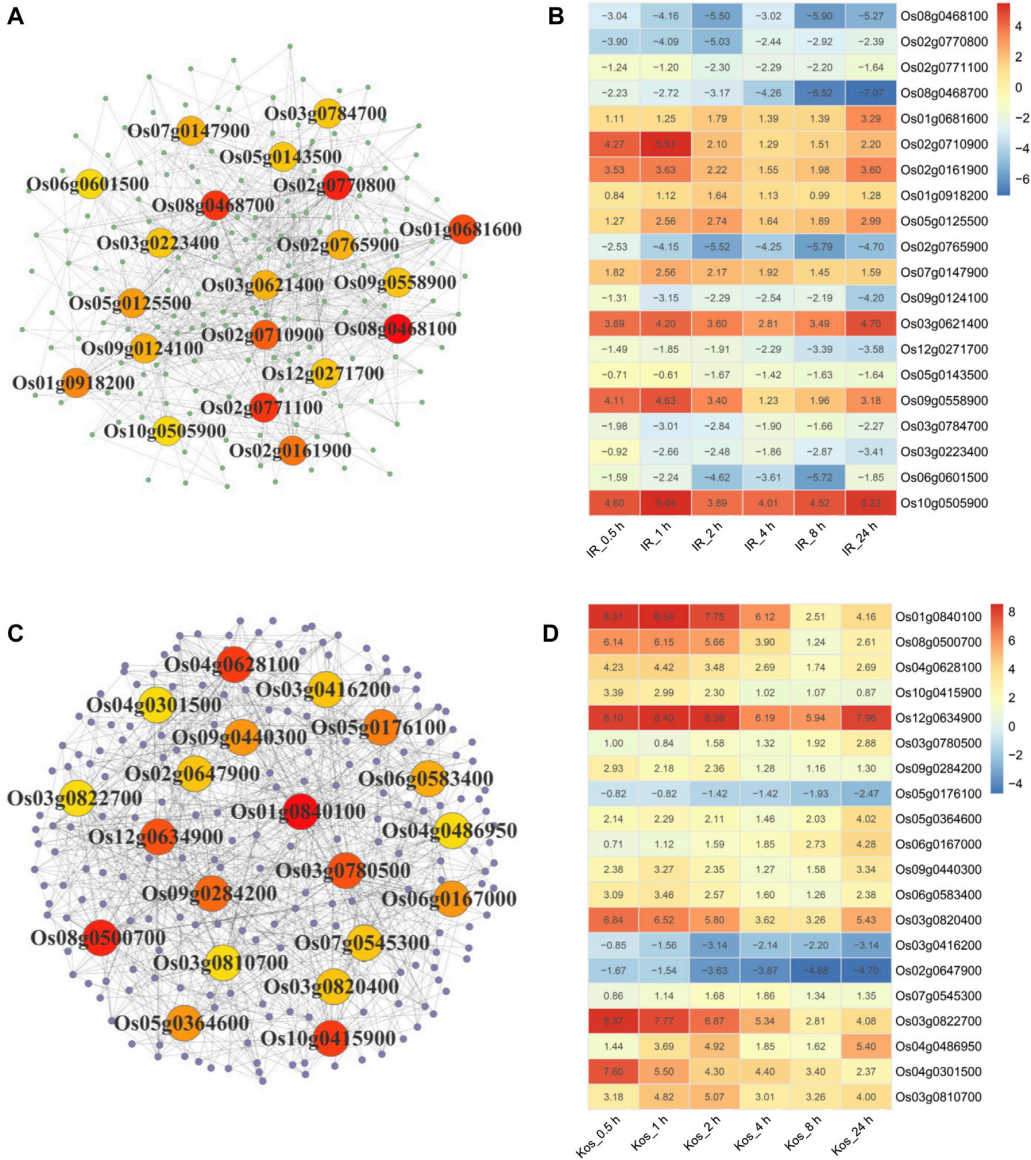
PPI analysis of IHR and KHR DEGs. Protein-protein interaction networks of IHR **(A)** and KHR **(C)** DEGs were constructed by STRING database. Each dot in the graph represents a gene, and the line indicates the relationship between the genes. The top 20 DEGs in the interaction network are calculated according to the degree, and the darker the color indicates the larger the degree unDir. The log_2_ Fold Change (log_2_ FC) of the top 20 DEGs in the IHR **(B)** and KHR **(D)** PPI network are represented in the heat map. The number indicates the log_2_ FC value of the DEGs at the corresponding time point.

We constructed PPI networks of IHR and KHR DEGs to facilitate further identification of genes located in core positions. The top 20 DEGs with the highest degree of protein interactions were shown in the PPI network of IHR and KHR (Figures 5A,C), while the expression patterns of these genes were depicted in the heat map (Figures 5B,D). There were 11 genes showing downregulation in IHR, while only 3 genes were downregulated in KHR. Three HSPs gene (*Os02g0710900*, *Os01g0840100*, *Os08g0500700*) were included in these core genes, all of which were upregulated relative to the control. In addition, we identified five genes related to nitrogen metabolism in the IHR, including *Os08g0468100* (*OsNIA1*), *Os02g0770800* (*OsNR2*), *Os08g0468700* (*OsNIA2*), *Os02g0765900* (*OsNIR2*) and *Os03g0223400* (*OsGSr*).

### 3.8 Analysis of CHR DEGs in two cultivars

Analysis of the DEGs of the two cultivars showed that there were 599 common DEGs, then the log_2_ FC value of these DEGs were shown in Supplementary Figure S3. After HS treatment, the CHR DEGs of the two cultivars showed similar expression patterns, implying that the response pattern of these genes to HS are the same in both cultivars. In addition, the number of upregulated genes were significantly higher than the number of downregulated genes in CHR DEGs.

**FIGURE 6:**
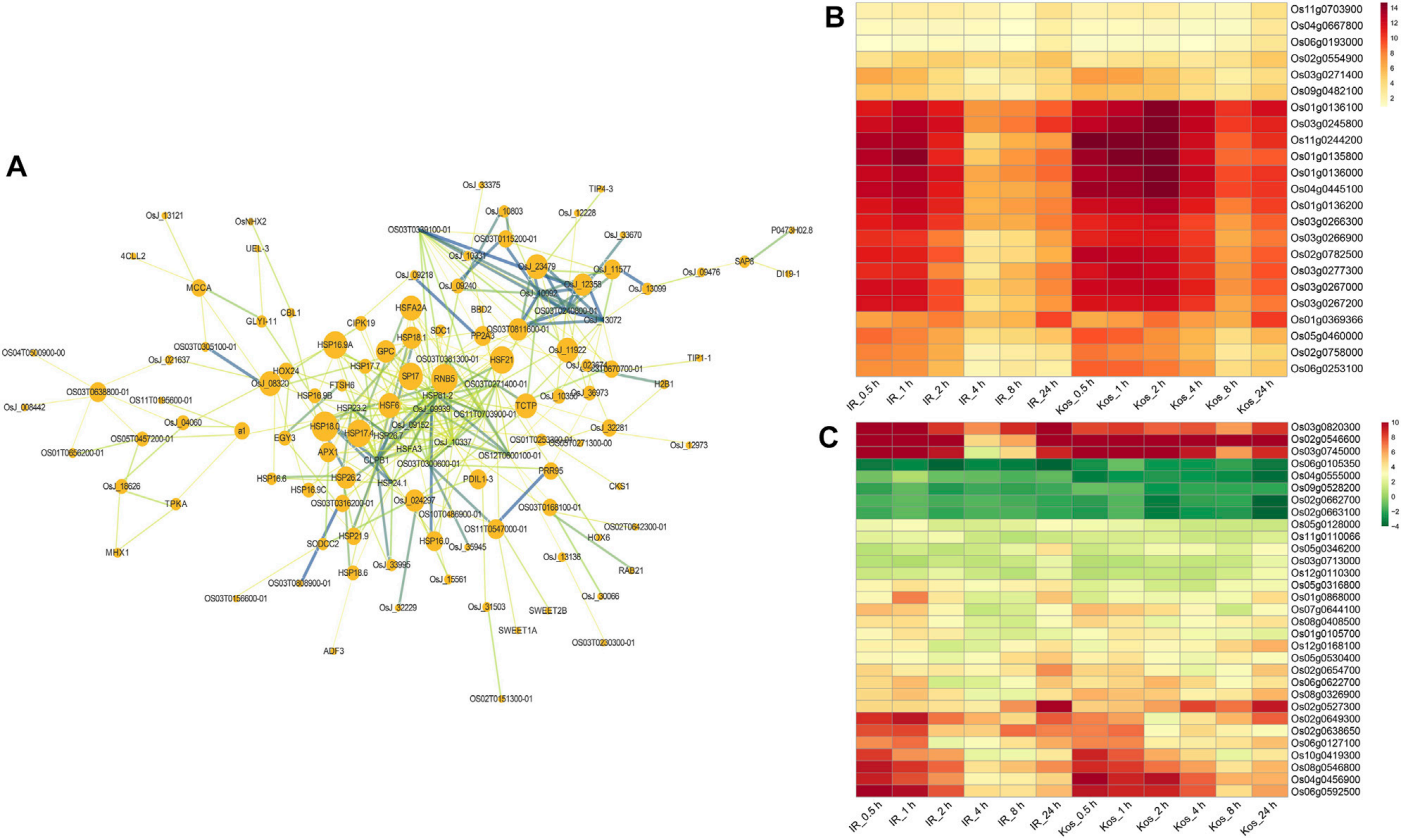
Analysis of CHR DEGs in two cultivars. **(A)** A PPI network for the CHR DEGs using the STRING database. Lines stand for relation of the target nodes. Color of lines stand for the distance of relationship between the nodes. Node size of nodes is proportional to the importance of protein. Heat map show the log_2_ FC values of genes in CHR DEGs belonging to the protein processing in endoplasmic reticulum **(B)** and DNA-binding transcription factor activity **(C)**, respectively.

As shown in Figure 6A, HSPs were the most abundant in the PPI network. Within the PPI network, it is worth noting that some key proteins may play key roles in the HS response, such as HSP81-2, CLPB1 (HSP100), OS11T0703900-01, OsJ_09939 (HSP70), OsJ_10337, HSP26.7, OsJ_09152, OS03T0271400-01, HSFA3, HSP23.2. In addition, some non-HSPs may also play important role in HS tolerance at seedlings, such as CIPK19, CBL1, APX1, a1, PP2A3, PDL1-3, HOX24, etc.

We performed GO and KEGG enrichment analysis of CHR DEGs. The results of GO enrichment analysis indicated that the DEGs of CHR were involved in heat acclimation, response to alcohol, regulation of programmed cell death, DNA-binding transcription factor activity, chaperone binding and Hsp90 protein binding, and so on (Supplementary Figure S4). Analysis of the KEGG pathway showed that the DEG of CHR were significantly involved in protein processing in endoplasmic reticulum, spliceosome, ubiquitin mediated proteolysis and RNA degradation (Supplementary Figure S5). We focused on the expression of DEGs in two pathways, protein processing in endoplasmic reticulum and DNA-binding transcription factor activity, which were significantly enriched. As shown in Figure 6B, there were 23 genes were enriched in the protein processing in endoplasmic reticulum pathway, and these included 18 HSP genes and 2 ubiquitin-conjugating enzyme genes, among others. Interestingly all genes that were enriched in the protein processing in endoplasmic reticulum pathway exhibit upregulation. A total of 31 DEGs were identified as belonging to the DNA-binding transcriptional activity pathway, of which 5 DEGs were downregulated (Figure 6C). Heat shock factors (Hsfs *Os08g0546800*, *Os02g0527300*, *Os03g0745000*, *Os10g0419300*), basic leucine zipper (bZIP) transcription factor (*Os06g0622700*, *Os07g0644100*), AP2/EREBP transcription factor gene (*Os02g0546600*, *Os02g0654700*, *Os02g0638650*, *Os05g0346200*, *Os05g0316800*, *Os01g0868000*, *Os06g0127100*, *Os08g0408500*), and TFIIIA-type zinc finger protein gene (*Os03g0820300*) were upregulated in both cultivars after HS treatment.

## 4 Discussion

Temperature increased, due to global warming, has become one of the most important factors constraining rice yield and quality (Zhao et al., 2017). Therefore, the identification of more beneficial genes involved in HS tolerance in rice is necessary for adaptation to extremely high temperature. In this study, we used two different subspecies of rice, and analyzed the 2,044 cultivars-difference core genes as well as 599 co-expressed core DEGs. The results may facilitate the discovery and annotation of important genes to rice under HS and provided a comprehensive responsive expression profile to HS. Further studies on these differentially regulated genes will provided insights into the differential HS responses of different rice subspecies.

It is commonly recognized that domestication and selection process led cultivated rice to different subspecies, namely *indica* and *japonica*, which represents two partially isolated gene pools and major source of genetic diversity of the rice germplasm (Smýkal et al., 2018). *Indica* and *japonica* rice have amount of genotypic variation, which makes them behave differently in face of stress. Variation of nitrate-transporter gene (*NRT 1.1B*) has led to the divergence in nitrate use (Hu et al., 2015). *bZIP73*, *OsAPX1*, *CTB4a* and *Ctb1* have a superimposed effect of dominant allelic variants that are important for adaptation to cold regions (Liu et al., 2018; Guo et al., 2020). Sequence variation in the *OsHMA3* promoter contributes to differential grain Cd accumulation between the two-rice subspecies (Liu et al., 2020). Historically, different rice production areas favored different subspecies, and the different climatic conditions in each production areas also lead to different adaptations to temperature among different subspecies (Raza et al., 2019). Many studies have shown that seedlings of *indica* and *japonica* rice have genotypic differences in response to HS, with *indica* rice having a higher temperature threshold than *japonica* rice, showing a basal or inherent thermal tolerance to HS (Khan et al., 2019; Dar et al., 2021). Our results show that tropical *indica* (IR64) exhibits greater HS tolerance relative to temperate *japonica* (Koshihikari). In addition, the number of consistently expressed DEGs in Kos was greater than the number of consistently expressed DEGs in IR, which suggests a greater effect of HS on Kos gene expression.

In the present study, the results of GO analysis of IHR and KHR DEGs exhibited two distinct HS response pathways. IHR DEGs were significantly enriched in chloroplast-related pathways, such as chloroplast envelope, chloroplast inner membrane, plastid inner membrane, photomorphogenesis, response to UV and red or far-red light signaling pathway, etc. Numerous studies have concluded that chloroplasts are essential for activating cellular HS signals and have also been proposed as sensors of HS. Processes associated with photosynthesis are susceptible to HS, including chlorophyll biosynthesis, CO_2_ assimilation, electron transport, photophosphorylation, and chloroplast membrane fluidity, which are the main metabolic processes that optimize carbon fixation and growth (Wahid et al., 2007; Allakhverdiev et al., 2008; Kmiecik et al., 2016; Sun and Guo, 2016; Hu et al., 2020). In addition, several confirmations have been made that retrograde signaling from chloroplasts can regulate the response of the nucleus to HS (Sun and Guo, 2016). To verify this opinion, we measured the contents of chlorophyll a and chlorophyll b in the leaves of the two rice cultivars after 24 h of HS treatment, and the results showed that the contents of chlorophyll a and chlorophyll b were significantly lower in Kos after HS treatment, while there was no change in IR (Supplementary Figure S7). Unlike IHR, HS mainly affects DEGs of KHR in the endoplasmic reticulum (ER) and mitochondria-related pathway. Both ER and mitochondria are associated with the HS tolerance in plants. The accumulation of unfolded or misfolded proteins due to HS can activate ER stress, which in turn affects redox metabolism and ROS production (Cao et al., 2022). Mitochondria-mediated lipid homeostasis is a buffer for maintaining flower development in rice under HS (Zhang et al., 2016). In conclusion, the unique HS response pathways of IHR and KHR may confer different HS tolerance in IR and Kos.

Notably, among the core genes of the PPI network constructed by IHR DEGs, we identified three nitrate reductase (NR) genes (*OsNIA1*, *OsNR2*, *OsNIA2*), one nitrite reductase (NiR) gene (*OsNIR2*) and one glutamine synthetase gene (*OsGSr*). Meanwhile, the pathways in which IRH DEGs are significantly enriched in GO and KEGG also contain the nitric oxide (NO) biosynthetic pathway and the nitrogen metabolism pathway, respectively. Furthermore, our data showed that exogenous application of 1 mmol/L nitrate reductase inhibitor (sodium tungstate Na_2_WO_4_) significantly reduced the heat stress tolerance of IR seedlings after 46 h of high temperature treatment at 45°C (Supplementary Figure S6). Sodium tungstate can inhibit NR activity in plants, which in turn reduces the production of NO (Freschi et al., 2010; Kolbert et al., 2010; Kaya et al., 2021). NO has been shown to be a key signaling molecule during plant development and different stress responses, NR is the most important source of NO, and controlling the balance of NO signaling molecules (Chamizo-Ampudia et al., 2017). The NR-deficient double-knockout mutants has reduced NO levels and nitrite concentration is one of the rates limiting factors (Planchet et al., 2005). NO synthesis in guard cells mediated by *NIR1* and *NIR2* in *Arabidopsis* mediates ABA-induced stomatal closure (Desikan et al., 2002). In addition, tobacco plants expressing the antisense NiR gene showed a significant accumulation of nitrite and a significant 100-fold increase in NO emission rate compared to the wild type (Morot-Gaudry-Talarmain et al., 2002). Salt stress induces the expression of the nitrate-responsive transcription factor *OsNLP2*, which directly or indirectly activates the expression of *OsNR1* and the ABA catabolism gene *OsABA8ox1*, leading to increased salt tolerance during rice seed germination (Yi et al., 2022). NO is actively involved in plant HS response by mitigating heat-induced oxidative stress, maintaining membrane integrity, and improving photosynthetic productivity (Parankusam et al., 2017). Xuan et al. (2010) showed that the nitrate reductase-deficient double mutant *nia1nia2* had lower survival ratio and NO levels under HS treatment compared with the wild type, which could be reversible by treatment with exogenous NO. NO can act synergistically with hydrogen sulfide at high temperatures to reduce H_2_O_2_-induced oxidative stress through the ascorbate-glutathione cycle and enhance HS tolerance in wheat (Iqbal et al., 2021). Overall, these results strongly suggest that NO biosynthetic pathway-related genes, especially nitrate reductase genes, may play a very important role in the IR64 response to HS.

Disruption of protein homeostasis is an important damage caused by HS to plants, which will lead to cell death and cytotoxicity. Plants threatened by HS will result in unfolding, misfolding, or denaturation of proteins, and plants will produce enough HSPs to counteract this. As important molecular chaperones, HSPs stabilize and degrade damaged proteins and promote HS-induced refolding of aggregated proteins (Müller and Rieu, 2016). Purified OsHsp17.4 and OsHsp17.9A proteins have chaperone activity that prevents their substrates from forming large aggregates, and heterologously expressed transgenic tobacco plants have better HS tolerance (Sarkar et al., 2020). In addition to HSPs, Hsfs are also important components of plants in response to HS and are responsible for triggering transcriptional cascades and activating downstream genes such as ROS scavenging enzymes, metabolic enzymes and HSPs. Rice *OsHSFA2dI* participated in the unfolded protein response under HS by alternative splicing (Cheng et al., 2015). *HsfA6a* mediates transcriptional regulation of the *HSP101* gene in rice (Singh et al., 2021). We identified a large number of genes encoding HSPs and Hsfs among the IHR KHR and CHR DEGs, including *hsp82A*, *HSP70*, *HSP26.7*, *OsHsfB2b*, *hsp82B*, *OsHSP24.1*, *OsHSP17.7*, *HSA32*, *HSP100*, *OsHsfA2a* and some uncloned genes containing heat shock protein typical domains, many of which are core genes in the PPI network. These genes have sustained expression for plants in response to HS and may be important candidate genes for HS tolerant breeding. Lin et al. (2014) showed that *HSP100* and *HSA32* interact and a positive feedback loop formed at the post-transcriptional level could extend the effect of heat acclimation at the seedling stage of rice. *OsHsfB2b* expression was strongly induced by heat treatment, but was barely affected by cold stress (Xiang et al., 2013). Overexpression of the small heat shock protein, *OsHSP17.7*, confers greater HS tolerance in rice (Murakami et al., 2004). Therefore, HSP and Hsfs play a central role in plants responding to HS, and may be good candidate genes for HS breeding in rice.

## Data Availability

The data presented in the study are deposited in the NCBI repository, accession number PRJNA917024.
