# Supplementary figures and images for: Comparative transcriptome analysis of the mechanism difference in heat stress response between indica rice cultivar “IR64” and japonica cultivar “Koshihikari” at the seedling stage
Source: Front Genet. 2023 Apr 21;14:1135577. doi: 10.3389/fgene.2023.1135577 (PMC10160441; doi:10.3389/fgene.2023.1135577)

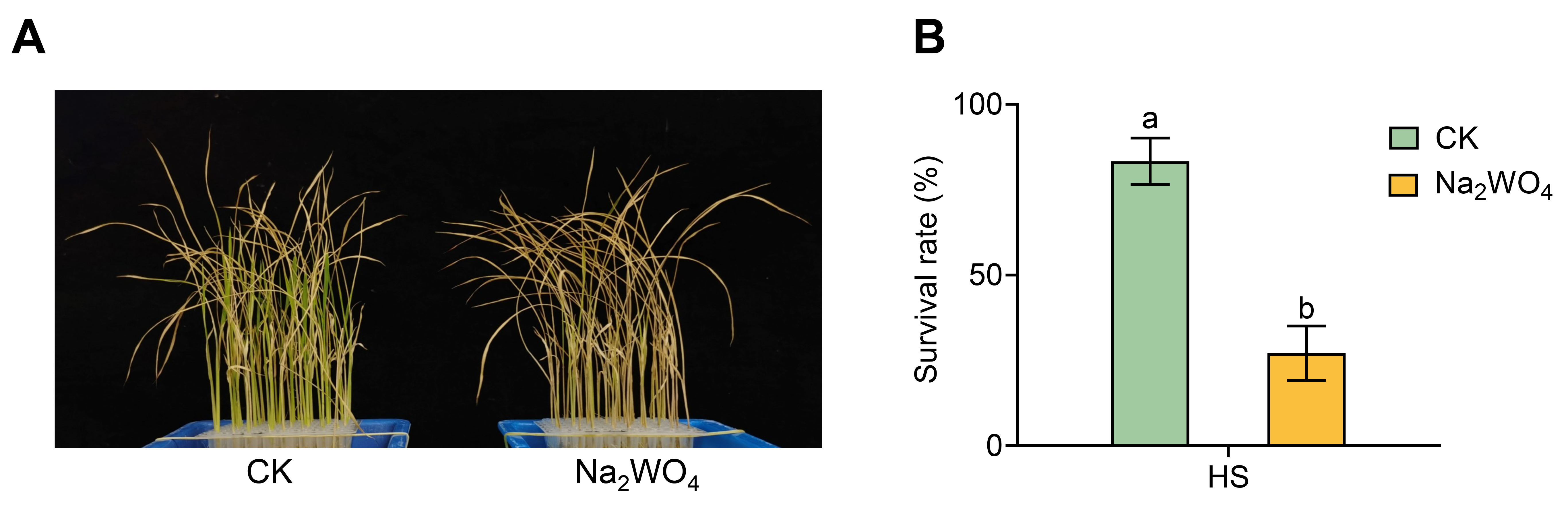

Supplement: Supplementary file 3 [file Image6.TIF]

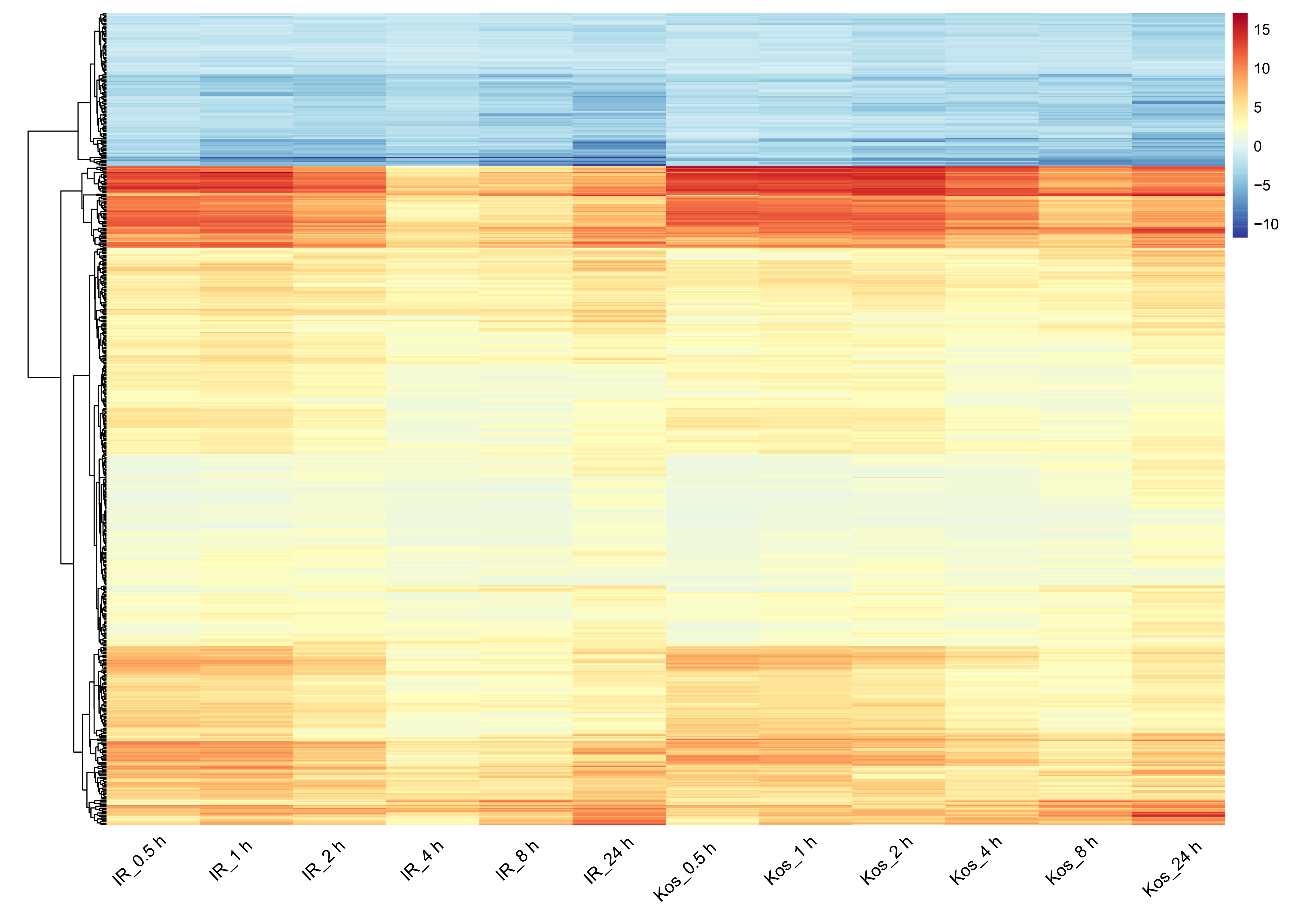

Supplement: Supplementary file 4 [file Image3.TIF]

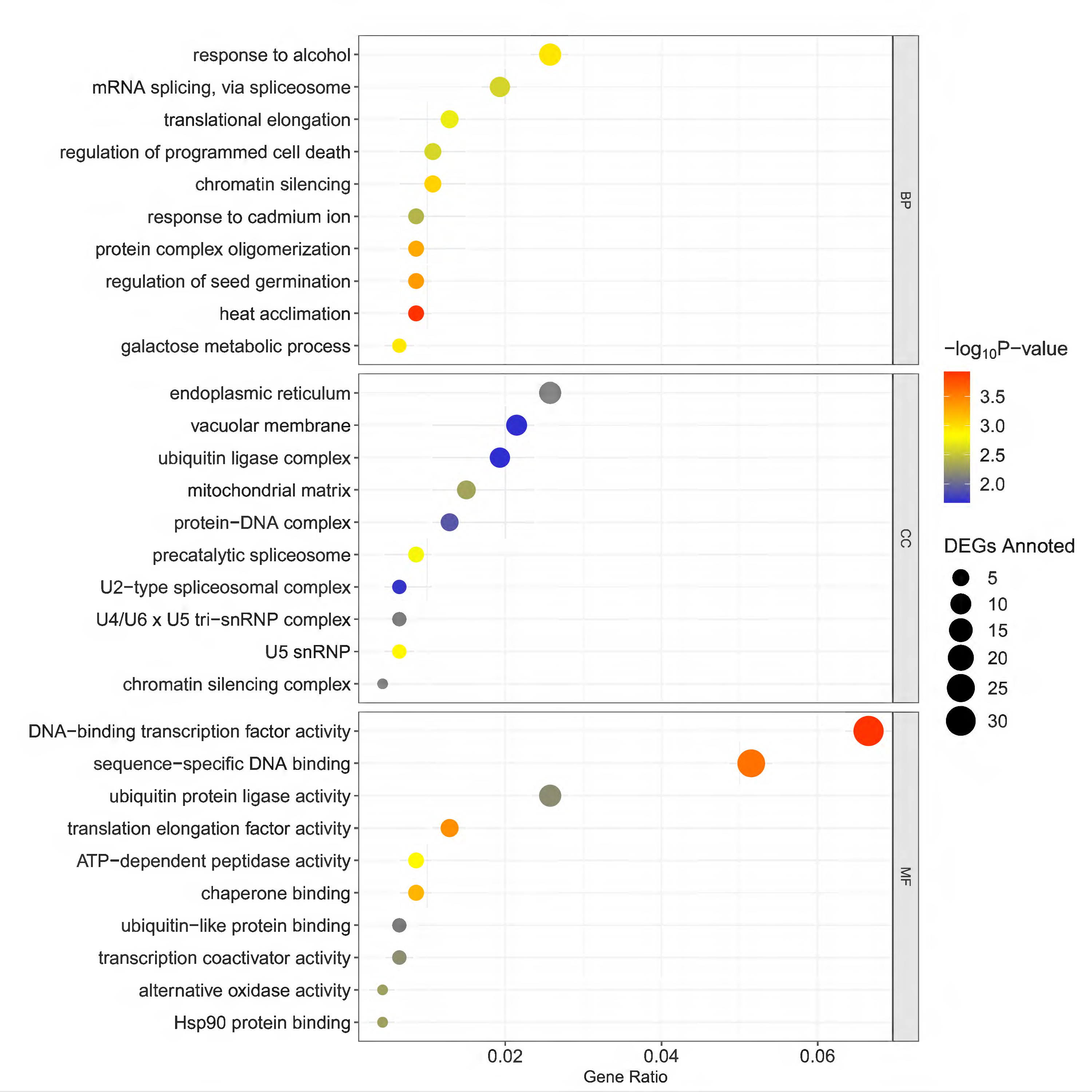

Supplement: Supplementary file 5 [file Image4.TIF]

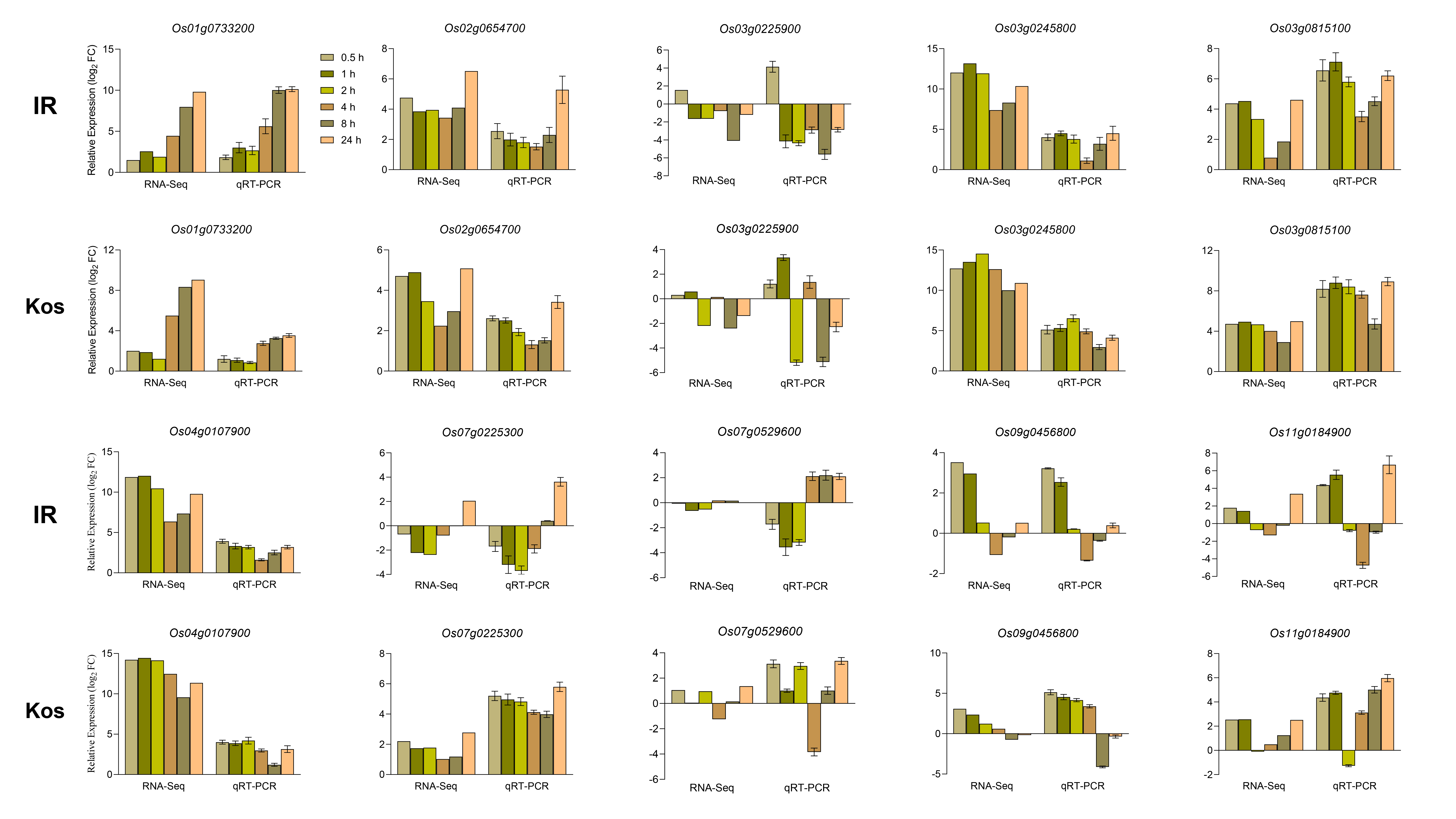

Supplement: Supplementary file 6 [file Image2.TIF]

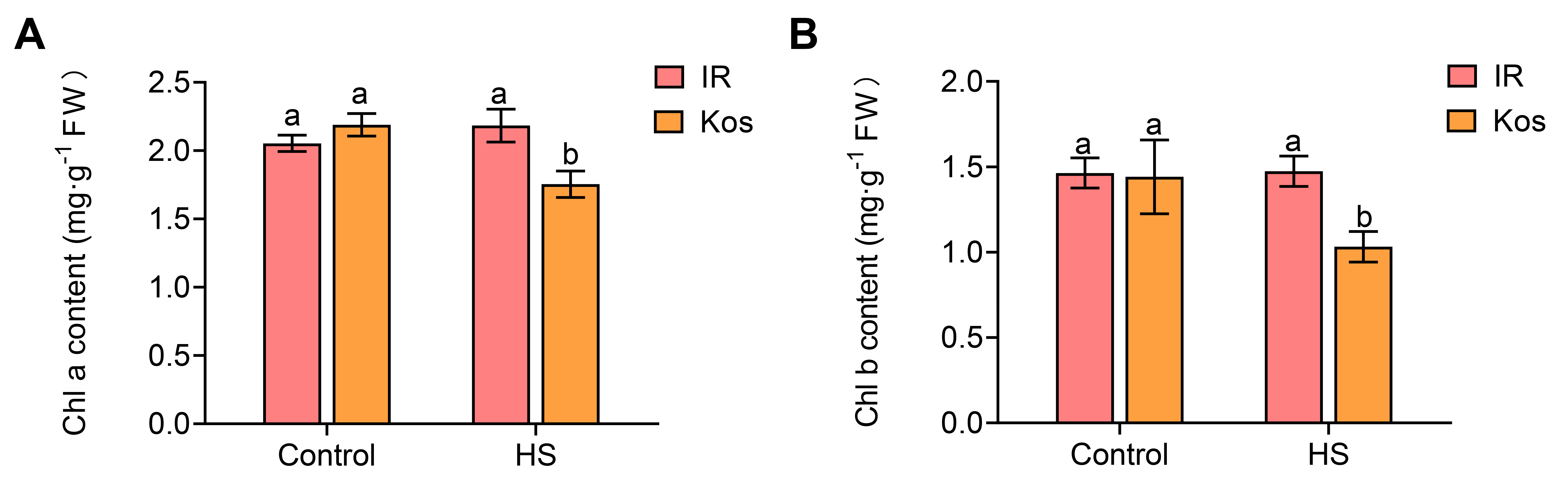

Supplement: Supplementary file 8 [file Image7.TIF]

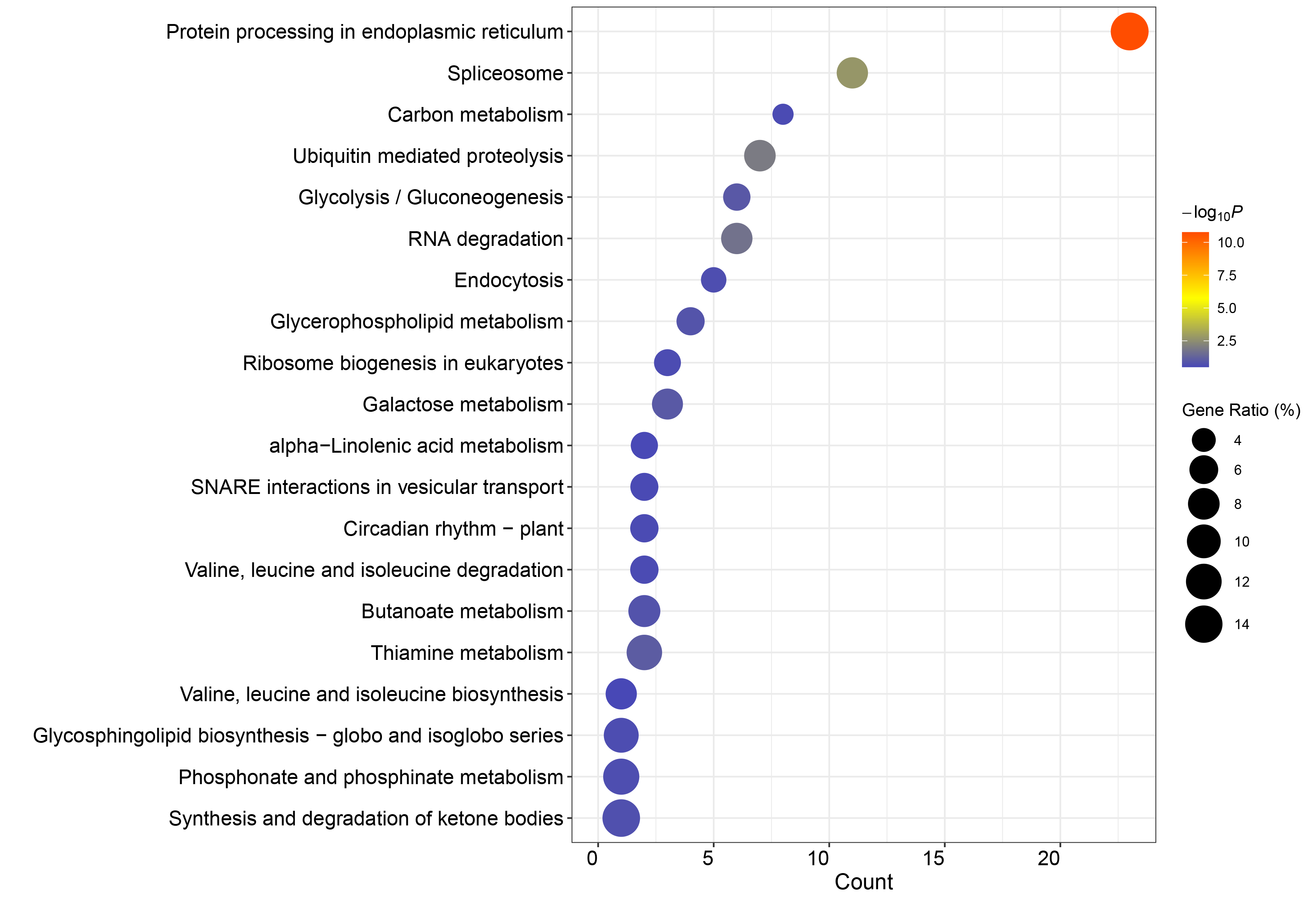

Supplement: Supplementary file 9 [file Image5.TIF]
